# Bone marrow lympho-myeloid malfunction in obesity requires precursor cell-autonomous TLR4

**DOI:** 10.1038/s41467-018-03145-8

**Published:** 2018-02-16

**Authors:** Ailing Liu, Minhui Chen, Rashmi Kumar, Maja Stefanovic-Racic, Robert M. O’Doherty, Ying Ding, Willi Jahnen-Dechent, Lisa Borghesi

**Affiliations:** 1 Department of Immunology, University of Pittsburgh School of Medicine, 200 Lothrop Street, Pittsburgh, PA 15261 USA; 2 School of Medicine, University of North Carolina at Chapel Hill, 60 Bondurant, CB 7000, Chapel Hill, NC 27599 USA; 3 Division of Endocrinology and Metabolism, Department of Medicine, University of Pittsburgh School of Medicine, 200 Lothrop Street, Pittsburgh, PA 15261 USA; 4 Department of Biostatistics, University of Pittsburgh Graduate School of Public Health, 130 DeSoto Street, Pittsburgh, PA 15261 USA; 5 University Hospital Aachen, Helmholtz-Institute for Biomedical Engineering, Biointerface Laboratory, Pauwelsstraße 30, 52074 Aachen, Germany

## Abstract

Obesity, a prevalent condition in adults and children, impairs bone marrow (BM) function. However, the underlying mechanisms are unclear. Here, we show that obese mice exhibit poor emergency immune responses in a toll-like receptor 4 (TLR4)-dependent manner. Canonical myeloid genes (*Csf1r*, *Spi1*, *Runx1*) are enhanced, and lymphoid genes (*Flt3*, *Tcf3*, *Ebf1*) are reduced. Using adoptive transfer and mixed BM chimera approaches we demonstrate that myeloid>lymphoid bias arises after 6 weeks of high-fat diet and depends on precursor cell-autonomous TLR4. Further, lean mice exposed to the TLR4 ligand lipopolysaccharide (LPS) at doses similar to that detectable in obese serum recapitulates BM lympho-myeloid alterations. Together, these results establish a mechanistic contribution of BM cell-intrinsic TLR4 to obesity-driven BM malfunction and demonstrate the importance of LPS. Our findings raises important questions about the impact of maternal obesity and endotoxemia to fetal hematopoiesis, as fetal immune precursors are also sensitive to TLR4 signals.

## Abstract (web summary)

Obesity can affect bone marrow cell differentiation and the generation of myeloid and lymphoid cells. Here, the authors show that diet and obesity, as well as low-dose lipopolysaccharide, can alter Toll-like receptor 4 signaling bone marrow cells to skew the myeloid-lymphoid homeostasis in mice.

## Introduction

Obesity, a condition of global impact, is associated with negative health outcomes including adverse events after bone marrow (BM) allogeneic transfer, increased risk of community-acquired and nosocomial infections, and influenza-associated mortality^1–5^. The prevalence of obesity not only in adults but also children, for whom obesity may be a life-long condition, prompts the need for a mechanistic understanding of BM malfunction^6^. In animal models, mice fed high-fat diet (HFD) have impaired emergency hematopoiesis following bacterial challenge, poor pathogen clearance, and increased mortality^7,8^. Several hematopoietic alterations are apparent even in homeostasis circumstances in obesity. For example, obese mice exhibit heightened BM myelopoiesis and abnormal frequencies of hematopoietic stem and progenitor cell (HSPC) subsets as well as gross thymic alterations^5,9–13^. Insight into the specific progenitor subsets impacted by obesity and the mechanistic underpinnings is highly desirable.

Two lines of evidence link obesity-associated immune cell dysregulation with the major innate immune sensor Toll-like receptor 4 (TLR4). First, obese animals deficient in TLR4 owing to germline ablation or to hematopoietic-specific deletion are substantially protected from inflammation, insulin resistance and type 2 diabetes^14,15^. Indeed, obesity-dependent TLR4 signals were shown to directly activate macrophage production of pro-inflammatory cytokines that contribute to inflammation and insulin resistance^11,15,16^. Reciprocally, TLR4 ablation shifts adipose tissue macrophages to an alternatively activated state associated with reduced inflammation^16^. Second, increased BM myeloid output in obese mice with frank diabetes requires TLR4^11^. TLR4-activated CD11c^+^ visceral adipose tissue macrophages release interleukin-1b (IL-1b) that, in turn, amplifies BM lineage^−^c-kit^+^Sca-1^−^ (LKS^neg^) myelopoiesis, essentially producing a feed-forward mechanism that reinforces inflammation^11^. BM changes are also apparent in the pre-diabetic stages of obesity but the responsible mechanisms have not been fully examined^11,12,17^. This is an important point as distinct mechanisms may contribute to BM alterations at different temporal stages of obesity-associated disease processes^5^. Intriguingly, recent studies demonstrate that BM cells express TLR4 and directly respond to TLR4 ligand in vivo^18−20^. Indeed, BM migratory HSPCs that circulate through blood and lymph are thought to patrol peripheral tissues before returning to BM. Following encounter with TLR ligand, these migratory HSPCs quickly boost the local supply of myeloid cells^21^.The implications of this BM TLR4 sensing pathway to obesity-associated BM malfunction have not been directly examined.

Also, unclear are the TLR4 ligands most relevant to BM malfunction in obesity. Lipopolysaccharide (LPS) and dietary saturated fatty acids are two TLR4 ligands recognized as potentially important in obesity-associated changes in metabolism and immune function^22–24^. For example, consumption of a high-fat meal but not a healthy control meal is sufficient to transiently increase plasma LPS in healthy human subjects and may underlie the chronically elevated concentrations of plasma LPS that characterize obesity^23^. Elevated serum LPS has also been attributed to chylomicron-mediated transport, increased gut permeability, and/or changes in gut microbial composition, which could further exacerbate obesity outcomes^22–24^. Notably, as little as 2 ng of LPS administered, i.p. rapidly depletes BM monocytes, suggesting exquisite sensitivity of the BM compartment to this potent TLR4 ligand^25^. Further, brief exposure to LPS activates BM precursor proliferation and myeloid-biased differentiation, whereas chronic LPS exposure drives persistent HSPC activation and a stem cell myeloid bias^18,20^. Saturated fatty acids common to high-fat diets, such as palmitate, have also been shown to directly activate TLR4 in macrophages^15^. Palmitic acid was furthermore recently shown to enhance BM myeloid colony formation and likely to bias immune cell development^13^. However, the in vivo importance of this pathway of TLR4 activation is controversial as saturated fatty acids are generally considered to be weak TLR4 ligands relative to LPS^11,15,26^.

Here, we use adoptive transfer and mixed BM chimera approaches to examine the impact of TLR4 to obesity-associated BM alterations, and begin to prioritize the importance of biologically relevant TLR4 ligands. In obese mice, modest changes in steady-state BM cellular composition give rise to impaired emergency hematopoiesis in a TLR4-dependent manner. In mixed BM chimeras, the TLR4-sufficient donor partner exhibits obesity-driven BM lympho-myeloid alterations, whereas the TLR4-deficient donor partner is protected, demonstrating a mechanistic contribution of BM cell-autonomous TLR4 activity. We further show that the BM myeloid>lymphoid bias that arises early in obesity depends on BM cell subset-autonomous TLR4. Moreover, chronic exposure to low-dose LPS recapitulates this key feature of obesity-associated BM damage. We conclude that cell-intrinsic TLR4 is required for BM malfunction in obesity.

## Results

### Modest BM perturbations in homeostasis amplified by stress

We first quantified BM progenitors in mice with diet-induced obesity and then examined the functional potential of defined precursor subsets. Mice were fed a 40 kcal% HFD or the nutrient-matched low fat control diet (NCD) starting at weaning, thereby ensuring uniformity of protein source, lard type and micronutrients. Following 16–18 weeks of exposure to HFD, obese mice had a 25% increase in body weight and 2–3 fold increase in fat stores, similar to previous findings (Supplementary Figure 1A)^27,28^.

**Fig. 1:**
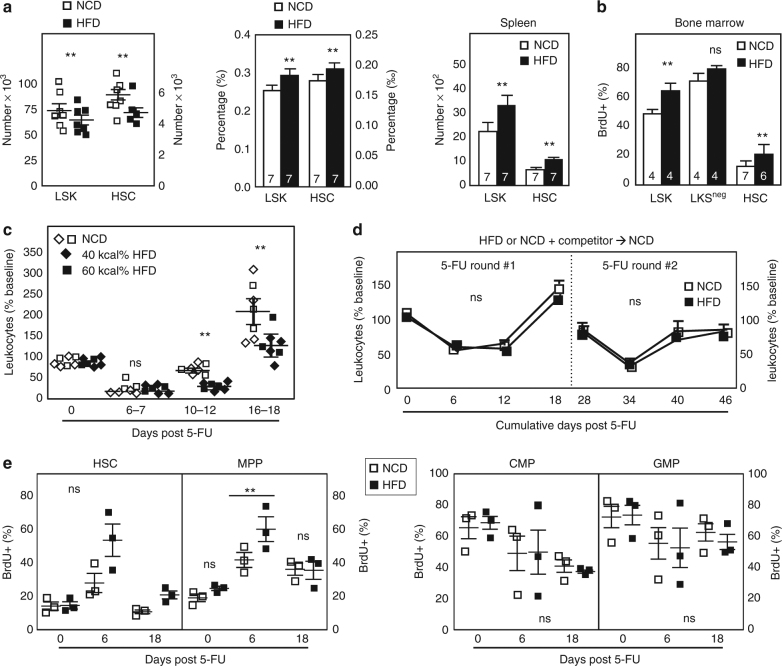
Obesity compromises hematopoietic potential. **a** Number and percentage of multipotent BM-LSK (lineage^−^Sca-1^+^c-kit^+^) and HSC (CD150^+^CD48^−^ LSK) in mice fed 40 kcal% high-fat diet (HFD) or nutrient-matched low fat control diet (NCD) for 16–18 weeks. In spleen, HSCs were identified using the CD150^+^flk2^−^ LSK definition as rare CD150^+^CD48^−^ LSKs were below the limit of detection by flow cytometry. Data represent the mean ± SEM of animals pooled from at least three independent experiments of NCD/HFD cohorts assayed in parallel for a total of *n *= 7 mice/group. **b** For analysis of BrdU incorporation, mice were injected twice daily with BrdU 48 h prior to killing. Following surface staining, cells were subsequently permeabilized for intracellular BrdU staining. Data are pooled from three independent experiments in which NCD/HFD mice were examined side-by-side with the total number of mice inset in the graph. **c** Mice fed the indicated high-fat diet for 16 weeks were exposed to the chemotherapy drug 5-fluorouracil (5-FU) and peripheral blood leukocyte rebound examined. Each diet (40 kcal%, diamond; and 60 kcal%,  square) was examined separately and the data pooled from a total of two independent experiments of 3–4 mice/group). **d** BM from B6 mice (CD45.2) fed either 40 kcal% high-fat diet or control diet for 16–18 weeks was separately mixed with competitor BM (CD45.1) and co-transferred to CD45.1/2 recipients. Following 16 weeks engraftment, a point at which hematopoiesis derives exclusively from donor HSCs, competitively engrafted chimeras were subject to two sequential rounds of 5-FU (day 0, 28) and peripheral blood leukocyte rebound of the CD45.2 donor partner examined. Data are pooled from two independent experiments (total *n* = 3–4 recipients/group). **e** Following 5-FU challenge as in **c**, BM subset BrdU incorporation was examined at the indicated time points. Data are pooled from 2–3 independent experiments of NCD/HFD mice assayed in parallel, and each symbol is an individual animal. Data are analyzed by Student’s t-test. Error bars represent s.e.m. **p < 0.05; ns, not significant

Absolute numbers of BM multipotent LSKs (lineage^−^Sca-1^+^c-kit^+^) as well as self-renewing hematopoietic stem cells (HSCs; CD150^+^CD48^−^ LSK) were reduced in BM of obese mice (Fig. 1a, phenotypic gating depicted in Supplementary Figure 1B). The frequency of BM-LSK and HSC progenitors marked by BrdU during a 48 h labeling window was increased 40–60% in obese animals (Fig. 1b). BrdU labeling performed in this way reflects total cell turnover within the labeling period inclusive of proliferation, survival and mobilization. Neither the frequency of cycling (S + G_2_M) progenitors as assessed by BrdU/DAPI nor the number of apoptotic cells as determined by staining with annexin V were detectably altered in obesity (Supplementary Figure 1C). However, both LSK and HSC subsets were numerically increased in the spleen of obese animals (Fig. 1a). In downstream BM subsets, no gross numerical alterations were detectable in myeloid precursors (common myeloid progenitor; granulocyte–monocyte progenitor; megakaryocyte–erythroid progenitor), lymphoid primed multipotent progenitors (LMPP), common lymphoid progenitors (CLPs), or precursors to the Group 2 innate lymphoid cells thought to limit adiposity (Supplementary Figure 1D). Previous studies report differences between lean and obese mice in the relative frequency of some of these subsets within flow cytometry gates and, under our experimental conditions, modest changes in percentages fell within standard error when calculated back to absolute numbers^5,11,12^.

Following hematopoietic ablation such as by chemotherapy, BM output increases several fold above baseline in order to meet a heightened demand for blood cell production^29^. We exposed mice to the chemotherapeutic 5-fluorouracil (5-FU), thereby forcing hematopoietic repopulation. Obese mice fed a moderate 40 kcal% (diamond symbols) or 60 kcal% (square symbols) HFD exhibit a ~50% reduction in blood leukocyte rebound compared to lean controls (Fig. 1c). Further, BrdU incorporation in obese HSC and MPP but not CMP and GMP was significantly increased following 5-FU (Fig. 1e). Emergency hematopoiesis in response to other acute stressors was also perturbed. Obese mice exhibited poor peripheral blood neutrophilia following exposure to an acute dose of the TLR4 ligand LPS (Supplementary Figure 2A), similar to previous reports of poor BM neutrophil efflux following challenge with live bacteria^7^.

The attenuated repopulation capacity of obese BM did not reflect permanent damage to precursor potential. Using a competitive transfer approach in which BM from obese mice or lean mice was separately mixed with competitor BM (lean) and transferred to lean recipients, we found that 5-FU responsiveness could be restored. Sixteen weeks post engraftment, a time point when hematopoiesis is exclusively derived from donor bone marrow, we subjected the BM recipients to 5-FU challenge. Donor BM derived from obese mice performed as well as donor BM derived from lean mice through not just one round but two sequential rounds of 5-FU ablation administered 4 weeks apart (Fig. 1d). Moreover, LSKs derived from obese versus lean donors had comparable levels of BrdU labeling within lean recipients, indicating restoration of normal patterns of LSK turnover (NCD: 60.5% ± 1.3, HFD: 58.9% ± 1.8, mean ± SEM, *n* = 3/group). Thus, we conclude that relatively moderate changes in steady-state hematopoiesis give rise to grossly impaired responses under challenge, and at least some of these defects can be reversed by removal of progenitors from the obese environment.

### HSPC functional and transcriptional defects in obesity

Hematopoietic rebound following insult or injury reflects the combined output of multiple progenitor subsets, therefore we examined the functional potential of discrete BM subsets in obesity: multipotent (HSC, MPP), myeloid (LKS^neg^), and B lymphoid (CLP, LSK). For these studies, we exploited serum-free, stroma-free *in vitro* cultures with the appropriate lineage-specific cytokines to evaluate the cytokine-induced differentiation capabilities of progenitors derived from obese mice under highly defined conditions.

**Fig. 2:**
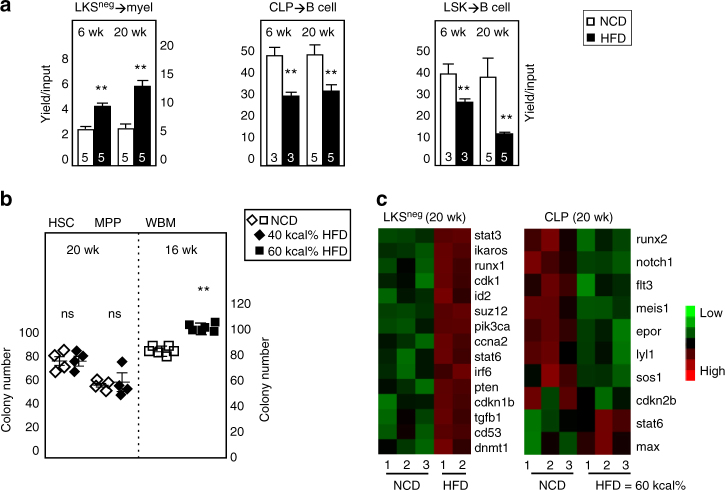
BM LKS^neg^ and CLP functional defects emerge early in obesity. **a** B6 mice were fed the indicated high-fat or nutrient-matched control diet for 6 or 20 weeks, after which defined BM precursor subsets were sorted for analysis of differentiation potential under stroma-free, serum-free conditions. lineage^−^c-kit^+^Sca-1^−^ (LKS^neg^), common lymphoid progenitor (CLP) and LSK subsets were assays in liquid cultures under myeloid- or lymphoid-supportive conditions for 8 or 12 days, respectively, after which cells were stained with antibodies to CD19, B220, Gr-1, or CD11b. Yield/cells input is shown. Bar graphs depict average ± SEM of data from two independent experiments of NCD/HFD mice assayed in parallel, with the total number of mice inset. **b** Sorted HSC (CD150^+^flk2^−^ LSK), MPP (flk2^+^ LSK), each at 100 cells per well, or 2 × 10^4^ BM cells (WBM) from NCD or HFD mice were cultured in Methocult and colonies enumerated on day 10. For scatter plots, data are pooled from three independent experiments of paired NCD/HFD mice. Each symbol is an individual animal. Bar graphs depict average ± SEM (duplicate experiments, total *n* = 4/group). **a**–**b** Data are analyzed by Student’s *t*-test with Bonferroni correction for multiple comparisons. Error bars represent s.e.m. **p < 0.05; ns, not significant. **c** NanoString expression analysis of canonical genes associated with lymphoid or myeloid potential in BM LKS^neg^ and CLP progenitors sorted from mice fed high-fat or control diet for 20 weeks. Each lane is a different mouse; one lane was lost due to an instrument technical problem

The lineage potential of both myeloid and lymphoid precursors isolated directly ex vivo from mice fed HFD for 20 weeks was changed as evidenced by a twofold increase in myeloid outgrowth (Gr-1^+^ or CD11b^+^) (Fig. 2a), consistent with previous reports of myeloid-biased BM^7,9,11–13^. Furthermore, sort purified CLP and LSK subsets from obese mice had a ~50% decrease in B lineage outgrowth (B220^+^CD19^+^) relative to lean controls (Fig. 2a). The enhanced outgrowth potential of LKS^neg^ progenitors did not extend to upstream self-renewing HSC or non-renewing MPP as assessed in Methocult cultures, which support granulocyte−macrophage colony formation (Fig. 2b). By contrast, whole bone marrow (WBM) from HFD mice had increased numbers of colonies relative to NCD animals, confirming past reports^12,13^ (Fig. 2b & Supplementary Figure 1E). No differences in colony size were detected (Supplementary Figure 1E). We then examined the RNA expression profiles of LKS^neg^ and CLP subsets isolated directly ex vivo using a NanoString panel containing probes to genes associated with lympho-myeloid differentiation, signal transduction and cell cycle activity (Fig. 2c). Select findings were validated and extended by RT-QPCR (Supplementary Figure 2B). LKS^neg^ progenitors from obese mice had increased expression of transcriptional regulators of myeloid differentiation (*Csf1r*, *Spi1*, *Runx1*), cell cycle activators (*Cdk1*, *Ccna2*) and signal transducers mechanistically linked to myeloid activity (*Stat3*, *Stat6*). CLPs had reduced expression of a hallmark cytokine receptor (*Flt3*), developmental stage-specific lymphoid transcription factors (*Tcf3*, *Ebf1*, *Ikzf1*), and several regulators of cell cycling (*Cdkn2b*, *Cdk1*, *Max*) (Fig. 2c & Supplementary Figure 2B). Downstream in the peripheral blood, a subset of obese mice exhibited spontaneously increased frequencies of Gr-1^+^ and CD11b^+^ cells at steady-state, a finding identical across animals fed the 40 kcal% or 60 kcal% high-fat diets. In contrast, B-cell frequencies were not detectably altered (Supplementary Figure 2C). Together, these findings show that HSPCs isolated from obese mice retain normal self-renewal activity in vivo and colony-forming activity in vitro (Figs 1d and 2b, respectively), whereas the myeloid potential of LKS^neg^ cells as well as the B-lymphoid potential of both LSK and CLP subsets are compromised at the transcriptional and functional levels.

### In vivo requirement for BM-intrinsic TLR4 in BM malfunction

**Fig. 3:**
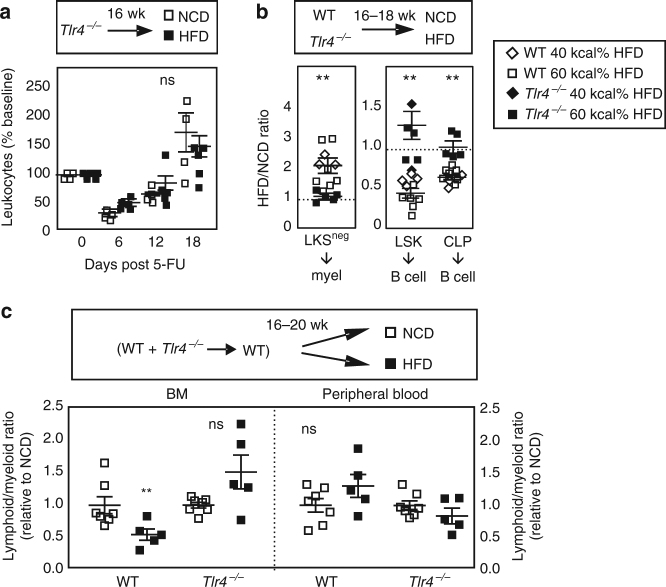
Myeloid>lymphoid BM bias requires TLR4. **a** Mice fed the 60 kcal% (square symbols) high-fat diet or matched control diet for 16 weeks were exposed to the chemotherapy drug 5-FU and peripheral blood leukocyte rebound tracked at the indicated time points. Data are pooled from two independent experiments of NCD/HFD mice analyzed side-by-side. **b** WT or *Tlr4*^−*/*−^ mice were fed 40 kcal% (diamond symbols) or 60 kcal% (square symbols) high-fat or matched control diet for 16–18 weeks after which sorted LKS^neg^ and CLP outgrowth was assessed in liquid assays as in Fig. 2. Data are pooled from four independent experiments of NCD/HFD mice examined in parallel. **c** WT (CD45.1) or *Tlr4*^−*/*−^ (CD45.2) BM was mixed and co-transferred to CD45.1/2 recipients, after which mice were rested for 1 week and then fed 60 kcal% (square symbols) high-fat or control diet for 16–20 weeks. (B220 + CD3)/Gr-1 is shown as lymphoid/myeloid ratio. Data are pooled from two independent experiments performed with different BM donors and 2–3 recipients/group. Each symbol is an individual animal. Data are analyzed by Student’s *t-*test. Error bars represent s.e.m. **p < 0.05; ns, not significant

To establish the in vivo role of TLR4 in impaired emergency immune responses in obesity, we compared 5-FU recovery patterns in WT and TLR4-deficient mice. In these experiments, TLR4-sufficient and TLR4-deficient age-matched, weight-matched mice were fed high-fat or control diet for 16 weeks. The weights were: lean B6 (31.6 ± 2.2), lean *Tlr4*^−*/*−^ (35.2 ± 1.6), obese B6 (46.4 ± 2.3), obese *Tlr4*^−*/*−^ (48.6 ± 1.4) (average grams ± SD; *n* = 6–8 mice/group). Despite weight gain, *Tlr4*^−*/*−^ mice fed HFD had no detectable changes in BM HSPC numbers or peripheral blood lympho-myeloid composition (Supplementary Figure 3A). Mice were then challenged with a single dose of 150 mg/kg 5-FU. In contrast to the poor hematopoietic rebound of WT mice fed HFD, the TLR4-deficient obese counterparts fed HFD for 16 weeks retained normal hematopoietic repopulation kinetics following 5-FU ablation (compare Figs 3a and 1c). Further, the obese TLR4-deficient mice were fully protected from both BM LKS^neg^ enhancement and CLP inhibition (Fig. 3b). These findings suggest a broader importance of TLR4 to BM malfunction in obesity than is currently appreciated.

TLR4 is expressed on BM precursors, on the stromal cells and mesenchymal stem cells (MSC) that form BM niches, and on mature immune cells in the periphery. To empirically determine whether the requirement for TLR4 signals is cell-intrinsic or cell-extrinsic we generated mixed BM chimeras. Equal numbers of BM from WT and *Tlr4*^−*/*−^ mice were mixed and co-engrafted to WT recipients. In this strategy, since both the WT and *Tlr4*^−*/*−^ cells are allowed to develop in the same cellular microenvironment, any change in the responses of the *Tlr4*^−*/*−^ donor counterpart indicates a cell-intrinsic effect. One week after engraftment, recipient mice were fed control or HFD for 16–20 weeks. While WT mice exhibited myeloid bias, we found that TLR4-deficient donor counterparts within the same recipients were protected (Fig. 3c). We conclude that BM-intrinsic TLR4 is required for lympho-myeloid malfunction in obesity. We also examined lympho-myeloid ratios in the periphery. As myeloid bias had not yet fully translated to blood to the point of reaching statistical significance, we could not make a definitive conclusion other than to note the trend toward myeloid production in WT but not *Tlr4*^−*/*−^ (Fig. 3c). A previous study demonstrated a key role for obesity-associated IL-1 in enhancing BM monocytosis in TLR4-sufficient CMP and GMP^11^. Our observation that *Tlr4*^−*/*−^-derived donor cells are protected from lympho-myeloid malfunction despite being in a WT cytokine microenvironment suggests that TLR4 signals are acting upstream of IL-1 signals.

### Chronic low-dose LPS recapitulates lympho-myeloid malfunction

**Fig. 4:**
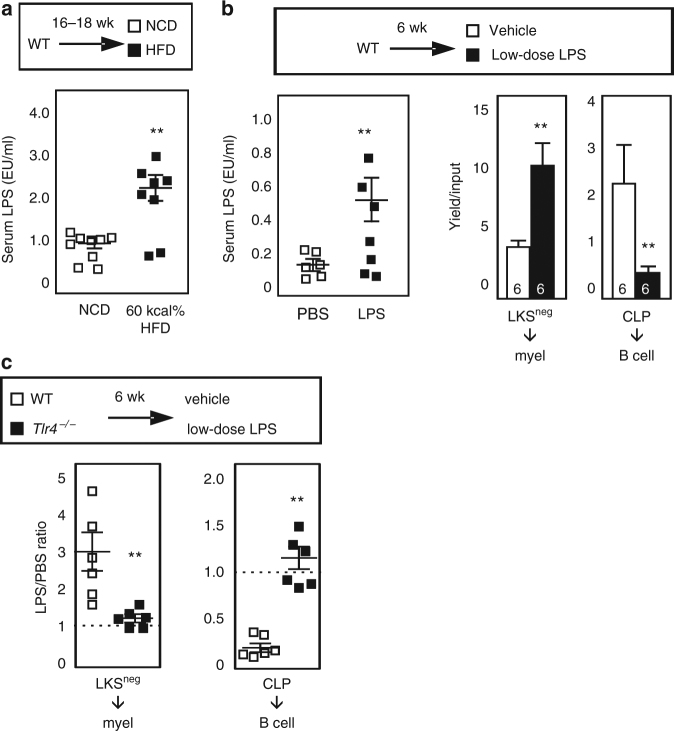
Chronic low-dose LPS administered at levels found in obese mice induces myeloid bias. **a** Serum LPS levels in B6 mice fed the indicated high-fat or control diet for 16–18 weeks. Data are pooled from 3 independent experiments in which NCD/HFD mice were analyzed side-by-side (total *n* = 8–9 mice/group). **b**, **c** WT or *Tlr4*^−*/*−^ mice were exposed to chronic low-dose LPS or PBS vehicle for 6 weeks after which serum LPS levels were assessed and the outgrowth potential of sorted LKS^neg^ and CLP subsets examined. Data are pooled from two to three independent experiments in which PBS and LPS mice were examined in parallel. Bar graphs depict average ± SEM, with number of mice inset. For scatter plots, each symbol is an individual animal. Data are analyzed by Student’s t-test. Error bars represent s.e.m. **p < 0.05

BM tissue is highly responsive to TLR4 ligand administered at low doses in vivo^25^. One characteristic feature of obesity in mouse and man is increased levels of serum LPS (i.e., low-grade endotoxemia), and we confirm this finding (Fig. 4a)^22–24^. We examined the potential of LPS, administered at doses physiologically relevant to obesity, to drive LKS^neg^ enhancement and CLP inhibition in lean mice. Following 6 weeks of LPS exposure at a dose of 6 μg i.p. every other day, serum LPS in lean mice was increased 2–4-fold, similar to the magnitude of increase over baseline observed in obesity (compare Fig. 4a, b**)**. Animal weights were not detectably altered by this low level of LPS (PBS: 26.9 ± 0.9, LPS: 26.1 ± 0.6; average grams ± SEM; n = 9 mice/group), similar to past reports^18,19^. Similarly, *Tlr4*^−*/*−^ BM HSPC or peripheral blood subsets were not detectably altered by LPS exposure (Supplementary Figure 3B). Although LPS concentrations within BM niches *per se* are difficult to formally quantify, LPS administered i.p. readily reaches BM^18^. Mice exposed to chronic LPS for 6 weeks had a 150% increase in LKS^neg^ myeloid outgrowth and an 80% decrease in CLP lymphoid outgrowth, similar to the alterations observed in obese animals (Fig. 4c). Further, both alterations were TLR4-dependent as expected. In addition to LPS, saturated fatty acids abundant in high-fat diets serve as TLR4 ligands. The identification of fetuin-A as a physical adapter between TLR4 and dietary saturated fats provides the opportunity to investigate the mechanistic role pathway in obesity-driven BM malfunction^30,31^. *Fetuin-A*^*+/*−^ mice are on the C57BL/6N background and we compared WT and *Fetuin-A*^−*/*−^ littermates following 6 weeks of exposure to HFD or NCD. While CLPs from obese WT mice had poor B lineage output, LKS^neg^ potential from these same animals was not increased, suggesting strain specificity in the impact of HFD on BM (Supplementary Figure 2D). CLPs from *Fetuin-A*^−*/*−^ mice were protected from HFD. We interpret these results with caution as WT mice gained more weight than *Fetuin-A*^−*/*−^ mice, and further experiments are needed to determine a causal role of Fetuin-A (Supplementary Figure 2E, 41% versus 14% weight increase, respectively). Together, these findings suggest a much broader role for TLR4 in obesity-associated loss of BM integrity than is currently appreciated, with TLR4 regulating not only myeloid potential but also B lymphoid potential and hematopoietic rebound.

### Early LKS^neg^ and CLP defects require cell-autonomous TLR4

**Fig. 5:**
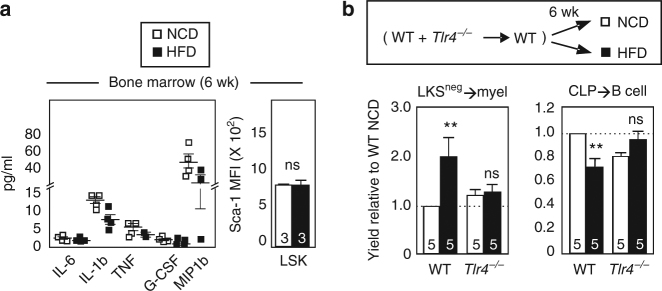
Requirement for BM cell-autonomous TLR4 in HFD-induced myeloid bias. **a** Left, cytokine levels in BM were assessed via multiplex ELISA following 6 weeks of 60 kcal% high-fat or control diet. NCD/HFD cohorts were examined in parallel and each symbol is a different animal. The thresholds of detection are: IL-6, 0.4 pg/ml; IL-1b, 5.4 pg/ml; TNF, 0.5 pg/ml; G-CSF, 0.5 pg/ml; MIP1b, 11.9 pg/ml. Right, Sca-1 mean fluorescence intensity (MFI) on BM-LSK. Data are analyzed by Student’s t-test. Error bars represent s.e.m. **b** WT (CD45.1) or *Tlr4*^−*/*−^ (CD45.2) BM was mixed and co-transferred to CD45.1/2 recipients, after which mice were rested for one week and then fed 60 kcal% high-fat or control diet for 6 weeks. At sacrifice, LKS^neg^ and CLP subsets from each donor were sorted for analysis of outgrowth potential as in Fig. 2. Data are pooled from two independent experiments performed with different BM donors and 2–3 recipients/experiment. Bar graphs depict average ± SEM, with number of mice inset. Data are analyzed by Student’s t-test with Bonferroni correction for multiple comparisons. **p < 0.05; ns, not significant

Several studies report BM myeloid bias after only 5–7 weeks of HFD, a stage of obesity that precedes gross diabetes, but the underlying mechanisms have not been examined^7,9^. We now show that both LKS^neg^ enhancement and CLP inhibition is detectable following only 6 weeks of HFD (Fig. 2a). At this relatively early stage of obesity, however, there was no detectable increase in protein concentrations of inflammatory cytokines in the extracellular fluid of BM including IL-1b, interleukin 6 (IL-6) or tumor necrosis factor (TNF) (Fig. 5a). These observations do not exclude the possibility that inflammatory cytokines are increased within local BM niches, at earlier time points not investigated or in other tissues where mobilized HSPCs might interact with inflammatory cytokines. Consistent with our findings, levels of Sca-1, an inflammation sensitive cell surface antigen, also were similar in BM of obese and lean mice after 6 weeks of HFD (Fig. 5a). The absence of readily apparent BM inflammation raised the possibility that inflammation-independent mechanisms are responsible for BM malfunction during early stages of obesity, which contrasts with the role of inflammation in perpetuating disease in established obesity^11^. To test the possibility that BM precursor TLR4 regulates BM development in early stages of obesity, we generated chimeras in which WT BM (CD45.1) was mixed with TLR4-deficient BM (CD45.2) and co-engrafted into WT hosts (CD45.1/2). One week after engraftment, chimeric animals were exposed to HFD or NCD for 6 weeks. Strikingly, LKS^neg^ and CLP subsets from the TLR4-deficient donor partner were protected from the BM myeloid>lymphoid bias in obesity while their WT progenitor counterparts remained susceptible (Fig. 5b). We note that the *Tlr4*^−*/*−^ CLP → B-cell yield differs slightly from WT in both the NCD and HFD groups, an outcome similar to that observed in another model of TLR4 stress^19^. These findings place emphasis on a direct cell-autonomous role for TLR4 in early stage BM malfunction as both WT and *Tlr4*^−*/*−^ progenitors were within the same HFD chimeric recipient. Thus, distinct mechanisms appear to initiate versus perpetuate BM damage in obesity. In contrast to late stage obesity in which BM myeloid bias is driven indirectly by TLR4 on peripheral inflammatory macrophages^11^, cell-autonomous TLR4 is required for early stage BM lympho-myeloid malfunction.

## Discussion

Our findings demonstrate a broader role for TLR4 in obesity-associated BM dysfunction than is presently recognized, and reveal a direct requirement for TLR4 on BM precursors. Specifically, cell-intrinsic TLR4 is required for LKS^neg^ and CLP malfunction during stages of obesity when gross BM inflammation is not readily detectable. We show that the TLR4 ligand LPS is elevated in the plasma of obese animals, and administration of exogenous LPS in lean mice recapitulates BM LKS^neg^ and CLP functional alterations. These findings reveal a new cell-intrinsic pathway by which TLR4 regulates BM LKS^neg^ and CLPs, in addition to the known role of TLR4 acting indirectly via inflammatory cytokines. Thus, TLR4 acts to regulate normal BM development via distinct cellular mechanisms during different temporal stages of obesity and our findings define the importance of one biologically relevant TLR4 ligand, LPS.

Long-term (LT)- HSCs sustain life-long hematopoiesis in adults. Here we show that although the normalized proportion of BM HSC/HSPC is increased in obesity, similar to past reports^11,12^, absolute numbers of BM HSCs are decreased. The realization that HSC/HSPC are being consumed by obesity changes our thinking about the impact of HFD and obesity on HSC/HSPC integrity, and allows us to connect those original studies with new directions emerging in the literature. Why are BM HSCs being lost? We found no detectable evidence of HSC apoptosis or change in cell cycle status. However, our observations of increased HSC BrdU labeling, an indicator of cell turnover, combined with findings of increased numbers of HSCs in spleen suggest that obesity may mobilize HSCs to the periphery. Indeed, a recent study demonstrated that HSPCs accumulate in adipose tissue LSK (AT- LSK) during HFD^32^. Further, AT-LSK are shown to be one source of the adipose tissue macrophages (ATM) that perpetuate inflammation and metabolic disease in obesity. These findings raise major questions about the originating tissue source of AT-LSK. Are BM-LSK being mobilized to adipose tissue? Splenic LSK may be another source of AT-LSK. Knowledge of the originating tissue source of AT-LSK is critical for developing effective intervention strategies. One way to experimentally address this question is through genetic barcoding approaches that permit fate tracking of single HSC clones^33,34^. Following adoptive transfer of marked HSCs and acquisition of steady-state hematopoiesis, hosts can be shifted to HFD and the bar-codes of AT-LSK, BM-LSK and SPL-LSK compared. It will be important to rigorously exclude potential contamination of LSK by myeloid progenitors (normally Sca-1^−^), as Sca-1 expression is induced by inflammatory cytokines^18,35^. This can be accomplished using the alternative markers CD150 and CD48 instead of Sca-1^35^. Another approach to identifying migratory HSCs is through parabiosis experiments in which the circulatory systems of CD45.1 and CD45.2 mice are surgically joined. Dutta et al.^36^ used this strategy to identify migratory chemokine receptor-2 (CCR2^+^) HSC that generated myeloid cells in a model of ischemic injury. The authors then employed nanoparticle-based siRNA knockdown to reduce CCR2 levels, dampen HSC mobilization and diminish splenic seeding. In obesity, CCR2 has been identified as one mediator of monocyte migration into adipose tissue but the importance of CCR2 during HSC recruitment has yet to be examined^37^. Intriguingly, Singer et al.^12^ demonstrated that HFD HSCs have enhanced potential to generate ATMs even following serial passage through NCD hosts before final transfer to HFD recipients. The mechanisms underlying the sustained capability of BM HSC to produce ATMs are unclear. One hint may come from studies of HSC dynamics in chronological aging in which myeloid-biased CD150^hi^ HSCs become overrepresented with age relative to CD150^int/lo^ HSCs^38^. Changes in BM HSC composition in obesity may lead to the preferential enrichment of particular migratory HSC subsets capable of infiltrating adipose stores and generating ATMs.

Nagareddy et al.^11^ were first to identify that ATM-derived IL-1 promotes monocytosis through effects on BM CMPs and GMPs in obesity. A recent study opens up the possibility that this paradigm may be extended to IL-1 effects on BM HSCs as well. Not only can BM HSCs directly respond to IL-1 but chronic IL-1 exposure was shown to direct myeloid-biased HSC differentiation in vivo^39^. These observations take on additional importance as obesity affects not just adults but also children and BM alterations may impact life-long immune function. In one model of sustained obesity in which mice were given HFD starting at 6–7 weeks of age and then maintained on HFD for another 25 weeks, BM cellularity was observed to initially contract and then to steadily increase^28^. We did not detect a pattern of BM contraction/expansion and the different experimental designs of our studies, including nutritional composition of HFD and age at which animals are first exposed to HFD, make the findings harder to directly compare. What is clear is that obesity is a prevalent condition, and multiple independent studies have shown dramatic alterations in the earliest BM precursors that replenish the immune system.

Here we show a BM cell-intrinsic role for TLR4 in LKS^neg^ and CLP malfunction following HFD. However, our studies do not preclude a role for obesity effects on stromal cell niches. Indeed, HFD has been shown to alter HSPC niches as well as bone architecture to the benefit of myelopoiesis^10,40^. HSPCs are found adjacent to BM sinusoids and in close proximity to mesenchymal stem cells (MSC). Short-term (6 week) exposure to HFD decreased the number of CXCL12-abundant reticular (CAR) cells and slightly increased the frequency of nestin^+^ BM MSCs^40^. This finding is important as different niches have different functional roles. For example, conditional ablation of CXCL12 impairs CLPs and B lymphopoiesis without apparent effects on HSCs^41^. Thus, obesity-associated loss of CAR niches, or alteration in CAR niche function, may reinforce myeloid>lymphoid biased hematopoiesis. Interestingly, TLR4 sensing by CAR cells is thought to regulate homeostasis by regulating the frequency of circulating monocytes. Following a pulse of low-dose LPS, CAR cells produce macrophage chemotactic protein that drives monocyte emigration from bone marrow in a matter of minutes^25^. By contrast, higher LPS doses combined with other inflammatory signals drive distinct MSC responses. MSC have been stratified into functional subtypes, pro-inflammatory MSC1 and immunosuppressive MSC2 subsets^42^. Whereas exposure to TLR4 ligand elicits the MSC1 phenotype and production of IL-6 and IL-8, other ligands such as TLR3 agonists direct the milder MSC2 phenotype^42,43^. Thus, in a unified model, physiological levels of circulating LPS establish a basal tone that guides the homeostatic MSC phenotype. LPS fluctuations of modest scope may elicited a self-limited wave of myelopoiesis, whereas sustained increases in LPS in tandem with other signals may drive inflammatory MSCs and corrupt BM niches. TLR3 agonist has been used to direct MSCs to an immunomodulatory phenotype in animal models of cardiac damage^44^. Further, a number of clinical trials for MSC-based therapies in graft tolerance, autoimmunity and cardiac repair are ongoing^45^. Similar approaches may be useful for repairing local damage to BM MSC niches during obesity and, more broadly, for reducing risk of obesity-associated morbidities including cardiovascular diseases^46^.

What is the biological source of circulating LPS in obesity? It is generally believed that LPS translocates from the gut to the bloodstream via two mechanisms. First, as part of normal lipid digestion, LPS absorbed from the gut is transported by chylomicrons^47^. Consequently, there is a transient postprandial rise in circulating LPS^48,49^. However, the amount of LPS in the bloodstream is significantly higher following consumption of HFD versus a control diet, and repeated daily HFD may lead to persistent endotoxemia^22,23^. Second, obesity-associated inflammation compromises the integrity of the gut barrier, causing “leaky gut” and microbial translocation to the bloodstream^22,24,50^. Mice fed HFD exhibit increased intestinal permeability as measured by the appearance of each fluorescent dextran and GFP-tagged *E. coli* in blood following oral gavage^51,52^. LPS in the bloodstream rapidly diffuses to BM eliciting changes in TLR4/MD2 complexes on HSPCs^20^. Recently, therapeutic administration of the gut-specific anti-inflammatory agent 5-aminosalicylic acid (5-ASA) was shown to alleviate inflammatory and metabolic aspects of obesity, including endotoxemia^24^. By reducing gut accumulation of innate immune cells and inflammatory cytokines, 5-ASA treatment improved intestinal barrier integrity, reduced serum LPS, and increased both glucose tolerance and insulin tolerance. Importantly, the beneficial effects on inflammation and metabolism were observed even when 5-ASA was used to treat established obesity. Another therapeutic approach is to reduce homing of leukocytes to the colon using integrin antagonists. The monoclonal antibodies vedolizumab (targets α4β7) and natalizumab (targets α4 integrin) that are being used clinically to treat Crohn’s Disease and Inflammatory Bowel Syndrome may have further application to obesity^53^. Indeed, beta7-integrin-deficient mice are protected from obesity-associated insulin resistance and metabolic disease^24^.

Fetal cord blood (CB) HSPCs express TLR4, and a next question is the extent to which fetal HSPCs are vulnerable to maternal endotoxemia^54,55^. Early developmental events in childhood influence health later in life and increasing evidence points to durable changes in offspring following maternal obesity^56^. In non-human primates, maternal HFD diet changes the fetal epigenome and transcriptome, and alters offspring microbiome through at least 1 year of life^57–59^. Fetal liver is a key site of hematopoiesis during ontogeny. Only recently has the implication of maternal obesity to fetal HSPC activity been examined. Offspring derived from murine HFD dams had reduced numbers of LSKs, altered ratios of lineage-positive subsets, and distinct reconstitution patterns when transferred to male but not female HFD recipients^60^. Like adult BM HSCs, stimulation of CB HSPCs with LPS has been shown to augment myeloid outgrowth in vitro^54^. Even more striking is the impact of LPS on CB HSCs in vivo. Using a humanized mouse model in which CB was engrafted into immune deficient murine recipients, we previously showed that chronic low-dose LPS leads to CB HSC exhaustion and myeloid>lymphoid bias^19^. In future studies, it will be important to distinguish direct versus indirect mechanisms of LPS action on CB HSPCs. One strategy for testing a cell-intrinsic role for TLR4 is through shRNA knockdown^61^. Equal numbers of control and TLR4-knockdown HSPCs derived from the same CB donor may be mixed for competitive analysis in vitro or in vivo. Observations of myeloid bias in the TLR4-sufficient but not TLR4-deficient donor partner would provide support for a direct effect of LPS. Few treatment options are available for managing the risks to the fetus of maternal obesity^6^. 5-ASA, the gut-specific anti-inflammatory therapeutic that has been used to alleviate metabolic disease has minimal side effects, at least in adult, and may be a good candidate for further testing in murine models of pregnancy in the context of obesity^24^.

In 2016, the International Agency for Research on Cancer (IARC) Handbook Working Group re-affirmed a significant association between obesity and cancer risk in adults and children^62^. A recent review article summarizes evidence of link between obesity and multiple cancers of blood cell origin^63^. The IARC also reported strong evidence for a causal link between persistent inflammation and the obesity-cancer risk but the precise mechanisms remain unclear. Persistent TLR4 signals in the BM of obese individuals may contribute to a permissive inflammatory environment. In a mouse model of genetic pre-disposition to spontaneous acute lymphoblastic leukemia (ALL) HFD mice progressed to frank ALL earlier than controls, suggesting that obesity may promote tumorigenesis^64^. Fortunately, at least some obesity comorbidities are reversible. In pediatric patients treated for ALL, obese children who subsequently attained normal weight during treatment had morbidity risks equivalent to individuals who had normal weight throughout^65^.

Together, our findings reveal a new mechanism by which obesity impairs BM integrity. We show that BM malfunction arises early in obesity and depends on precursor-intrinsic TLR4. We further show that at least some BM perturbations can be recapitulated by persistent exposure to LPS at levels physiologically relevant to obesity. Obesity is an urgent health problem among adults and, increasingly, children. Interventions that reduce circulating LPS or dampen TLR4 signals may improve BM integrity in obese individuals.

## Materials and methods

### Mice

Four-week-old male C57BL/6J mice on the CD45.2 (Stock No: 000664) or CD45.1 (Stock No: 002014) background were fed nutrient-matched diets with 40 kcal% fat (Harlan TD.96001) or 60 kcal% fat (Research Diets D12492), starting at weaning, whereas control mice received respective lean diets matched for protein source, ratio of lard to corn oil (Harlan TD.110340 or Research Diets 12450B, respectively). Protein, vitamins, and minerals are equivalent on the basis of kcal density. Some cohorts using the same 60 kcal% and control Research Diets were purchased directly from The Jackson Laboratory (#380056 and control 380050). For chronic LPS exposure, 4-week old female C57BL/6J mice received 6 μg LPS or vehicle, i.p., for 6 weeks as described except that doses were administered every other day instead of daily^18^. Fetuin-A heterozygotes, a kind gift of Dr. Willi Jahnen-Dechent, were maintained on the original the C57BL/6N background by interbreeding. Mice were housed in a specific pathogen-free/SPF facility. Permission was granted to perform animal experiments by the Institutional Animal Care and Use Committee at the University of Pittsburgh School of Medicine.

### Flow cytometry and BrdU

Cell surface and intracellular staining was performed as described^19^. Primary anti-mouse Abs included lineage biotin (NK1.1 (PK136, dilution 1/100), CD11b (M1/70, dilution 1/400), CD19 (MB19-1, dilution 1/100), B220 (RA3-6B2, dilution 1/800), CD3 (145-2C11, dilution 1/100), TER-119 (TER-119, dilution 1/200), and Gr-1 (RB6-8C5, dilution 1/800)), Sca-1 FITC (D7, dilution 1/400) or APC (D7, dilution 1/200) or Cy5PE (D7, dilution 1/400), c-Kit PE (2B8, dilution 1/50), or Cy5PE (2B8, dilution 1/100), CD19 Cy5PE (MB19-1, dilution 1/50), B220 APC (RA3-6B2, dilution 1/800), CD115 PE (AFS98, dilution 1/400), CD135 PE (A2F10, dilution 1/50), CD150 Cy7PE (mShad150, dilution 1/50), CD48 APC (HM48^−^1, dilution 1/100), CD14 FITC (Sa2^−^8, dilution 1/25), CD45.1 PE (A20, dilution 1/100), CD45.2 FITC (104, dilution 1/400), ST-2 bio (RMST2-2, dilution 1/50). Secondary reagents were streptavidin-Cy7PE (dilution 1/800), -Cy7APC (dilution 1/800) or -Pacific Blue (dilution 1/400). Flow cytometry was performed on a 5 laser, 16 detector LSR II or a 5 laser 18 detector LSR Fortessa, and cell sorting was performed on a 5 laser, 17 detector Aria. Data were analyzed with FlowJo software (Tree Star). BrdU incorporation and annexin V staining was performed as we have previously described^66^. For BrdU, mice were injected i.p. with 600 μg BrdU or PBS at 12 h intervals, and 48 h after the first injection bone marrow was isolated. Cells were stained for surface markers followed by intracellular staining with anti-BrdU antibodies using the BrdU flow kit (catalog number: 556028) according to manufacturer’s instructions (BD Biosciences).

### Emergency hematopoiesis

Mice were injected i.p. with 150 mg/kg body weight 5-FU. Peripheral blood was analyzed prior to treatment and at the indicated time points thereafter by flow cytometry^66^. For acute LPS treatment, mice were injected i.p. with 1 μg/g body weight LPS or PBS control once a day for 2 days and were killed on day 3, as described^66^.

### Murine reconstitution chimeras

C57BL/6 male mice (6–8 weeks of age) served as hosts and also a source of competitor BM as described^19^. Donor and host/competitor were distinguished with CD45 alleles. Hosts given a lethal (900 rads) dose of gamma radiation were engrafted with a total of 2 × 10^6^ BM cells (donor + competitor) i.v. via tail vein. Following 1 week of engraftment, recipient mice were fed the HFD or NCD indicated in each figure legend.

### Morphometric and metabolic indicators of obesity

Body fat composition and liver triglycerides were determined as described^27^. For glucose tolerance testing, 6-h fasted mice were injected i.p. with 1.5 g/kg glucose and glucose was measured from venous blood with a glucose monitor.

### Cell culture

For serum-free liquid culture assays, sorted LKS^neg^, CLP or LSK subsets were cultured with X-VIVO 15 medium (Biowhittaker) supplemented with 10% BSA (StemCell Technologies) and either IL-7, flt3 ligand and stem cell factor (lymphoid) or M-CSF (myeloid) cytokines as we have done^66^. At harvest as indicated in the figure legend, cells were stained with antibodies to B220, CD19, CD11b, or Gr-1. For in vitro colony-forming assay, sorted HSC or MPP or WBM were cultured in MethoCult M3434 (StemCell Technologies) and colony-forming potential was assessed on day 10^66^.

### Gene expression analysis

RNA was extracted using an RNeasy Micro Kit per the manufacturer’s instructions (QIAGEN) and reverse transcribed into cDNA with Superscript III Reverse Transcriptase (Invitrogen) using oligoDT primers. For NanoString analyses, total RNA was hybridized and quantified with the nCounter Analysis System (NanoString Technologies) using a custom codeset (Supplementary Table 1). RNA was hybridized per manufacturer’s instructions and data were normalized to five housekeeping genes: tbp, G6pdx, Polr1b, Gusb, and HPRT using manufacturer’s nSolver software. For quantitative real-time PCR, reactions were performed in triplicate using TaqMan probes (Invitrogen). Expression levels were calculated for each gene relative to beta actin and expressed as the fold difference relative to control.

### Statistics

Statistical significance of differences between group means (*p* < 0.05) was established using Student’s *t*-test for pairwise comparisons. Multiple comparisons were performed using paired *t*-tests with the Bonferroni step down correction of *p*. Error bars on graphs reflect standard error of the mean, with the number of mice indicated in each figure.

### Data availability

The authors declare that the data supporting the findings of this study are available within the article and its supplementary information files, or are available upon reasonable requests to the authors. All Nanostring data have been deposited in the Gene Expression Omnibus (National Center for Biotechnology Information) under accession number GSE109269.

## Electronic supplementary material

Supplementary Information
