# Supplementary material for: Bone marrow lympho-myeloid malfunction in obesity requires precursor cell-autonomous TLR4
Source: Nat Commun. 2018 Feb 16;9:708. doi: 10.1038/s41467-018-03145-8 (PMC5816016; doi:10.1038/s41467-018-03145-8)
Supplement: Supplementary file 1 — Supplementary Information [file 41467_2018_3145_MOESM1_ESM.pdf]

Supplementary Figure 1

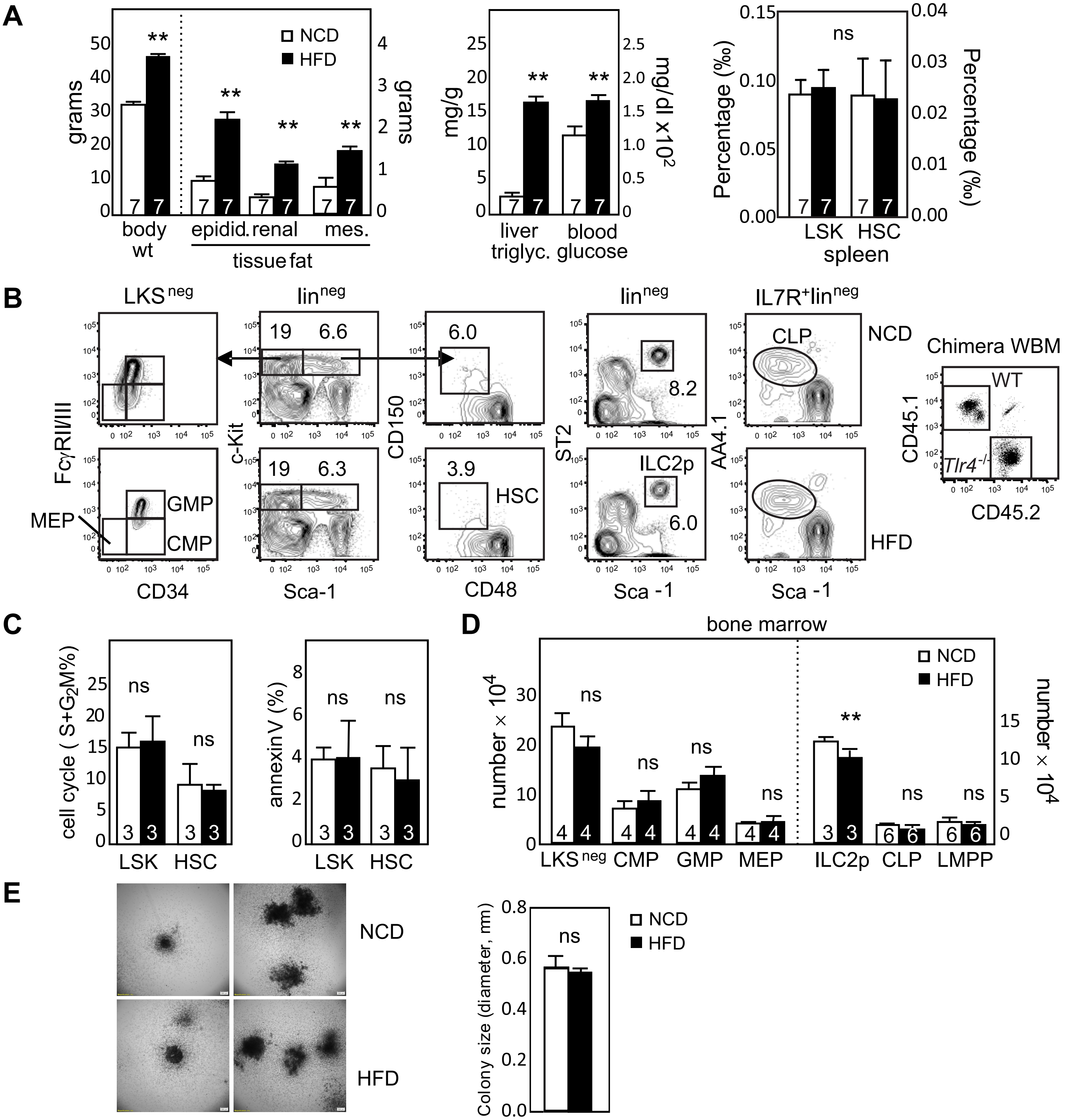

Supplementary Figure 1. Morphometric and immunological profile of obese mice. (A) WT animals fed 40 kcal% high fat diet or nutrient-matched low fat control diet for 16-18 weeks were weighed at sacrifice and analyzed for fat depots (Left panel). Liver triglycerides and blood glucose were assessed in parallel (Middle panel). The percentage of spleen LSK and HSC were also assessed (Right panel). Data represent mean ± SEM of mice pooled from three independent experiments for a total of n=7 mice/group. (B-D) Representative flow cytometry gating of BM and absolute number of depicted subsets. Phenotypic markers were LSK (lineage^{-}Sca-1^{+}c-kit^{+}), LKS^{neg} (lineage^{-}c-kit^{+}Sca-1^{-}), HSC (CD150^{+}CD48^{-} LSK), CMP (CD34^{+}FcγRII/III^{lo}), GMP (FcγRII/III^{+}CD34^{+}), MEP (FcγRII/III^{-} CD34^{-}), pILC2 (ST2^{+}Sca-1^{+}lin^{-}), CLP (AA4.1^{+}Sca-1^{lo}IL-7R^{+}lineage^{-}), and LMPP (flk2^{bright} LSK). Cell cycle status was assessed by BrdU/DAPI staining and apoptotic frequency was measured by Annexin V staining. Bar graphs depict average ± SEM with number of mice inset. A, C, D, data are analyzed by Student's t-test. **p<0.05; ns, not significant. (E) Representative images of day 10 Methocult colonies from NCD and HFD WBM cells. Images are from one experiment representative of two independent experiments each of which included n=2 mice/group with colonies quantified in triplicate wells for a total of n=6 data points, average ± SEM.

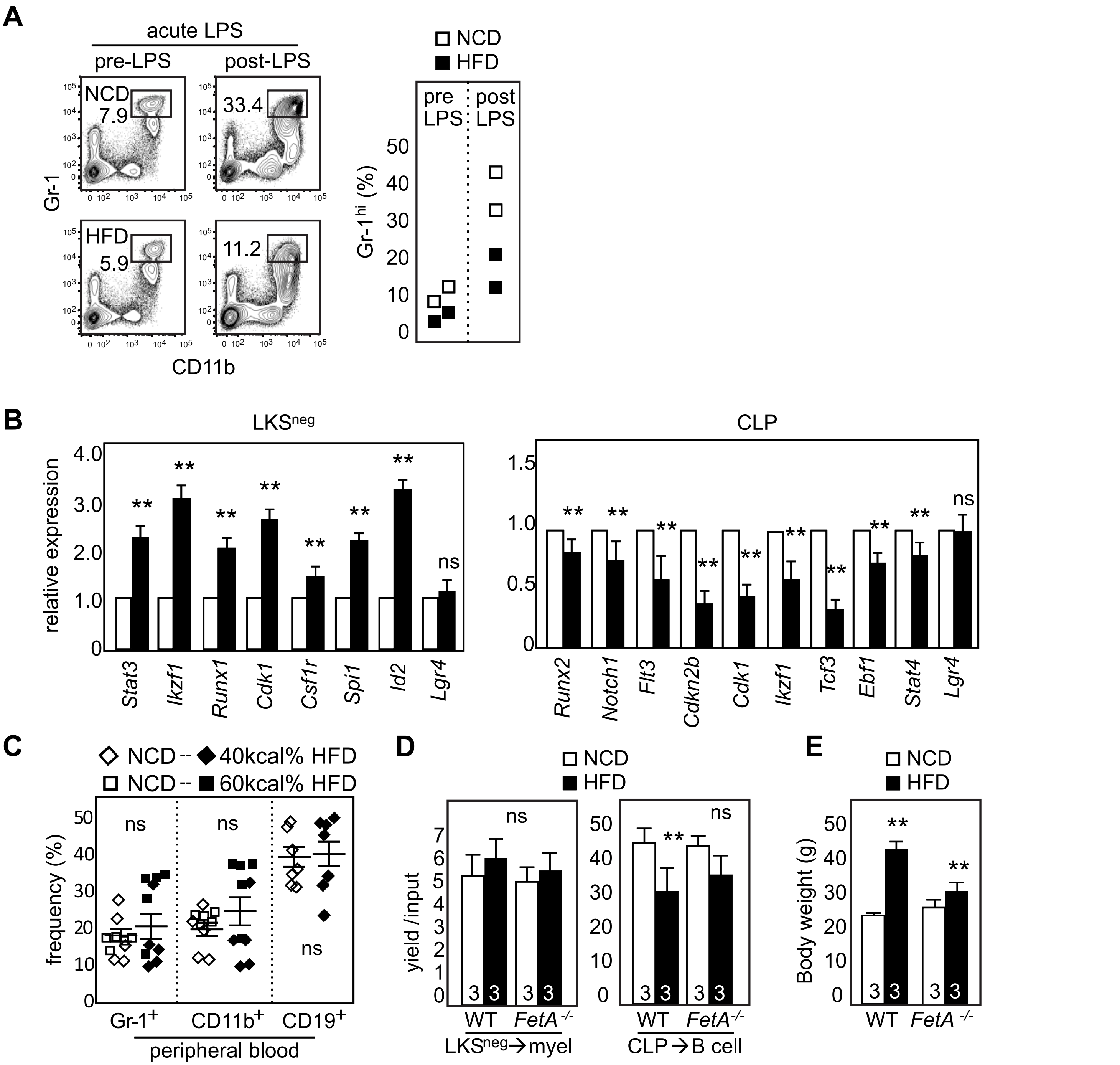

Supplementary Figure 2. Functional and gene expression profile of obese mice. (A) After 16 weeks of exposure to 60 kcal% high fat or control diet, WT mice were challenged by exposure to acute LPS and the frequency of pro-inflammatory Gr-1^{hi} neutrophils in peripheral blood calculated. Each symbol is an individual animal. (B) RT-QPCR analysis of BM LKS^{neg} (*left*) or CLPs (*right*) from mice fed 40 kcal% or 60 kcal% high fat diet or the respective nutrient matched control diets. Bar graphs depict average ± SEM of 3 independent sorts. (C) Peripheral blood composition of mice fed 40 kcal% or 60 kcal% high fat diet for 16-18 weeks. Each symbol is an individual animal. Data are pooled from 3 independent experiments. (D-E) WT or *Fetuin-A* $^{-/-}$ mice (C56BL/N background) were fed 60 kcal% high fat or control diet for 6 weeks, after which defined BM precursor subsets were sorted for analysis of differentiation potential under stroma-free, serum-free conditions. Weight in grams following HFD. Bar graphs depict average ± SEM with number of mice inset. Data are representative of two independent experiments of paired NCD/HFD cohorts. B-E, data are analyzed by Student's t-test. Error bars represent s.e.m. **p<0.05; ns, not significant.

Supplementary Figure 3

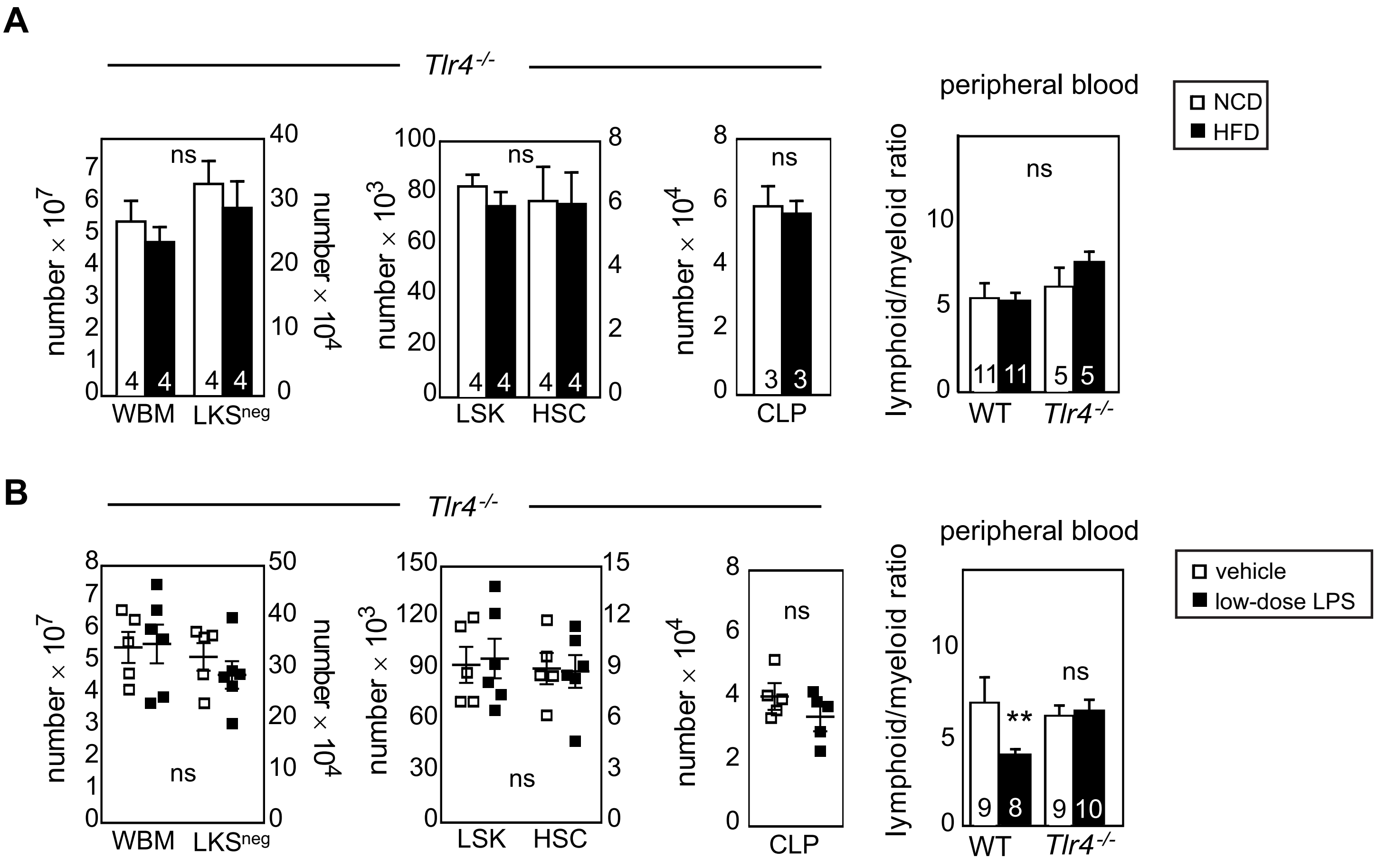

Supplementary Figure 3. BM subset cellularity in *Tlr4*$^{-/-}$ mice exposed to HFD or low-dose LPS. The indicated BM and peripheral blood subsets from *Tlr4*$^{-/-}$ mice fed 60 kcal% high fat diet or nutrient-matched low fat control diet for 16 weeks (A) or exposed to chronic low-dose LPS or PBS vehicle for 6 weeks (B) were quantified. The lymphoid/myeloid ratio, *far right*, is shown as (B220+CD3)/Gr-1. Bar graphs depict average ± SEM with number of mice inset. For scatter plots, each symbol is an individual animal. Data are pooled from two to four independent experiments of NCD/HFD analyzed side-by-side. Data are analyzed by Student's t-test. Error bars represent s.e.m. **$p<0.05$; ns, not significant.

Supplementary Table 1. NanoString CODESET

	Gene	Accession	Position	Target Sequence	Tm CP	Tm RP	Flags	HUGO Gene	NSID (internal identifier used by NanoString)
1	Actb	NM_007393.1	816-915	CAGGTCATCACTATTGGCAACGAGCGGTT	81	79	HK;X	Actb	NM_007393.1:815
2	Ahr	NM_013464.4	1328-1427	CTGCAGACATACTTCACTGTGCAGAATCC	83	82		Ahr	NM_013464.4:1327
3	Akt1	NM_001165894.1	899-998	GCCATGAAGATCCTCAAGAAGGAGGTCAT	83	83		Akt1	NM_001165894.1:898
4	Akt2	NM_001110208.1	2505-2604	CCGCTCGGCCCCTTTGGATGGCTCCGTTC	86	85		Akt2	NM_001110208.1:2504
5	APC	NM_007462.3	646-745	CCATGGGGTCATTCCCAAGAAGAACATTT	83	81		Apc	NM_007462.3:645
6	Bad	NM_007522.3	1147-1246	TTCGAGGCCTTAGGAAAAAAAAAGAGGAT	83	82		Bad	NM_007522.3:1146
7	Bax	NM_007527.3	736-835	CATAAATTATGACATTTTCCTGGGATGAAT	82	81		Bax	NM_007527.3:735
8	Bcl11a	NM_016707.2	524-623	CCGCCTTCCCCTTCTCCCATCGAGATGAA	82	81		Bcl11a	NM_016707.2:523
9	Bcl11b	NM_001079883.1	3561-3660	TCCGGGAGGTGGATGTACAGCGGATTACA	79	82		Bcl11b	NM_001079883.1:3560
10	Bcl2	NM_009741.3	1845-1944	GGCCTTCTTTGAGTTCGGTGGGGTCATGT	85	87		Bcl2	NM_009741.3:1844
11	Bmi1	NM_007552.4	3355-3454	TCTCGAGGTTTTCATGGTGTTACCTAAGA	78	80		Bmi1	NM_007552.4:3354
12	Brd3	NM_001113573.1	2691-2790	ACCAGCCTCTCCCTTAGTAACTTGAAGGA	82	80		Brd3	NM_001113573.1:2690
13	Casp8	NM_009812.2	1464-1563	TTTCATTCAGGCTTGCCAAGGAAGTAACT	82	84		Casp8	NM_009812.2:1463
14	Casp9	NM_015733.4	1676-1775	ATAACTGTCCTGCTAAGATAGGATTTTGAA	83	83		Casp9	NM_015733.4:1675
15	Cbx2	NM_007623.2	2081-2180	TTAGTTGTGTGGTTGGGCTCAGGGACCCT	82	82		Cbx2	NM_007623.2:2080
16	Cbx8	NM_013926.1	1046-1145	TGACTAACCTGGAGAAAGTGGTCGTTACA	79	82		Cbx8	NM_013926.1:1045
17	Ccna1	NM_007628.3	376-475	ACGTGGGTTTGATATCTATATGGATGATC	82	83		Ccna1	NM_007628.3:375
18	Ccna2	NM_009828.2	1281-1380	ACTGGATATACCCTGGAGAGTCTTAAGCC	83	79		Ccna2	NM_009828.2:1280
19	Ccnb1	NM_172301.3	2137-2236	TCATAGTAGCTCTTCCAGGGGTGTGCTTT	84	86		Ccnb1	NM_172301.3:2136
20	Ccnb2	NM_007630.2	281-380	CCAAAGTACCAGCTCTGCCCACCAAAGTG	79	82		Ccnb2	NM_007630.2:280
21	Ccnb3	NM_183015.3	3325-3424	TTTGAATCCAGAACAGACAATTCCAGTGC	80	79		Ccnb3	NM_183015.3:3324
22	Ccnc	NM_016746.3	141-240	GCTGGCTGGTGCATTTGTATCAGGGCAAG	81	80		Ccnc	NM_016746.3:140
23	Ccnd1	NM_007631.1	2001-2100	GGAGGGGTTCTAATGGAATGGATGGGGA	82	81		Ccnd1	NM_007631.1:2000
24	Ccnd2	NM_009829.3	1666-1765	CCAACCACATGACTGAACCATTTTGGATG	79	82		Ccnd2	NM_009829.3:1665
25	Ccnd3	NM_007632.2	1312-1411	GCCATGATGGTCAGAGAAATACAAACAGG	83	80		Ccnd3	NM_007632.2:1311
26	Ccne1	NM_007633.2	886-985	ATGATGATGAAGGCCCTTAAGTGGCGTCT	81	82		Ccne1	NM_007633.2:885
27	Ccne2	NM_001037134.1	614-713	CATTCTGACCTGGAACCACAGATGAGGTC	78	78		Ccne2	NM_001037134.1:613
28	CD34	NM_001111059.1	561-660	CTGATTATTCGCCTAATAATAGCAGCTTTG	79	80		Cd34	NM_001111059.1:560
29	CD48	NM_007649.4	371-470	TGCGTGAAACTGAGAACGAGTTGAAGATA	82	82		Cd48	NM_007649.4:370
30	CD52	NM_013706.2	363-462	CATCAGTAGCGAGAGACATCCCAACCCAC	86	86		Cd52	NM_013706.2:362
31	CD53	NM_007651.3	2301-2400	ATGGCATCAGTTATTCTGGGTCAAAAGTT	78	82		Cd53	NM_007651.3:2300
32	CD55	NM_010016.2	1059-1158	ACAGTTAAAGTTTCAGCAACCCAGCATGT	81	82		Cd55	NM_010016.2:1058
33	Cdk1	NM_007659.3	2539-2638	CCAGATCTCTGTTGGGAAGTAACTGGGTA	82	81		Cdk1	NM_007659.3:2538
34	Cdk2	NM_016756.4	1469-1568	TGCTGCCACTGTTTTGTGAACTAATGAAC	83	83		Cdk2	NM_016756.4:1468
35	Cdk4	NM_009870.3	495-594	CCTCCACCGGGCCTGCCGGTTGAGACCA	84	81	X	Cdk4	NM_009870.3:494
36	Cdk5	NM_007668.3	78-177	GGAAGGCACCTATGGAACTGTGTTCAAGG	88	87		Cdk5	NM_007668.3:77
37	Cdkn1a	NM_007669.4	1671-1770	AATACCGTGGGTGTCAAAGCACTTAGTGG	82	82		Cdkn1a	NM_007669.4:1670
38	Cdkn1b	NM_009875.4	2017-2116	AGGAAAAAAAGGACACTTGTAGAGTAAGTG	79	79		Cdkn1b	NM_009875.4:2016
39	Cdkn1c	NM_009876.3	1241-1340	CTGGGACCTTTCGTTCATGTAGCAGGAAC	82	80		Cdkn1c	NM_009876.3:1240
40	Cdkn2a	NM_001040654.1	567-666	CCAATCCCAAGAGCAGAGCTAAATCCGGC	83	78		Cdkn2a	NM_001040654.1:566
41	Cdkn2b	NM_007670.4	1041-1140	AAATGGGAAACCTGGAGAGTAGATGAGAG	83	81		Cdkn2b	NM_007670.4:1040
42	Cdkn2c	NM_007671.2	266-365	GCCAGGGGGGACCTAGAGCAACTTACTA	82	82		Cdkn2c	NM_007671.2:265
43	Cdkn2d	NM_009878.2	156-255	GAGGTCCGCCGCCTTCTTCATCGGGAGC	82	82		Cdkn2d	NM_009878.2:155
44	Cdkn3	NM_028222.1	279-378	TCAGAAAGATACAGAAGAACTAAAGAGCT	82	82		Cdkn3	NM_028222.1:278
45	Cebpa	NM_007678.3	1969-2068	AGGAGGACACGGGGACCATTAGCCTTGT	82	82		Cebpa	NM_007678.3:1968
46	Cebpb	NM_009883.3	1148-1247	CGGGCACCGCGCGCACGCACCTGCACAG	88	87		Cebpb	NM_009883.3:1147
47	Csf1r	NM_001037859.2	1039-1138	CAAGATAACTATTATAAAAAAGTCCGGGC	80	81		Csf1r	NM_001037859.2:1038
48	Ctnnb1	NM_007614.2	2976-3075	TTGGTCGAGGAGTAACAATACAAATGGAT	79	78		Ctnnb1	NM_007614.2:2975
49	Dnmt1	NM_010066.3	2381-2480	CTCGGTCATTCCAGATGATTCCTCCAAAC	80	80		Dnmt1	NM_010066.3:2380
50	Dnmt3a	NM_007872.4	7161-7260	TCCAGAGAGTAGAAGAAAATTCCTGGGCC	82	82		Dnmt3a	NM_007872.4:7160

	Gene	Accession	Position	Target Sequence	Tm CP	Tm RP	Flags	HUGO Gene	NSID (internal identifier used by NanoString)
51	Dnmt3b	NM_001003960.3	2313-2412	CCCATGCAATGATCTCTCTAACGTCAATCC	79	80		Dnmt3b	NM_001003960.3:2312
52	Dtx1	NM_008052.3	2019-2118	AACACCTCAAGAAAAGCAAGAATCCTGAG	82	83		Dtx1	NM_008052.3:2018
53	Dtx4	NM_172442.3	3616-3715	CCTTTGCCAGCCCATCTCGTAATAAACTTG	81	78		Dtx4	NM_172442.3:3615
54	Ebf1	NM_007897.2	891-990	CATAGGGGACAATTTCTTTGATGGGTTAC	78	83		Ebf1	NM_007897.2:890
55	Epor	NM_010149.3	295-394	TCTGTGCTTCACCCAACGCTTGGAAGACT	82	83		Epor	NM_010149.3:294
56	Erg	NM_133659.2	993-1092	AGCCATCTCCCTCTGCAGTGCCCAAAACT	85	85		Erg	NM_133659.2:992
57	Esr1	NM_007956.4	737-836	ACTCGCTACTGTGCCGTGTGCAATGACTA	83	83		Esr1	NM_007956.4:736
58	ETS1	NM_001038642.1	741-840	GAAAGAGGATGTGAAACCATATCAGGTTA	80	78		Ets1	NM_001038642.1:740
59	ETS2	NM_011809.2	3285-3384	TCAGTTTATGTACAGTGGAGCCACATGAC	79	78		Ets2	NM_011809.2:3284
60	Etv3	NM_012051.2	3451-3550	TGAGCTGACGTGGATCACTTGAGGCTGTC	81	82		Etv3	NM_012051.2:3450
61	Etv6	NM_007961.3	3860-3959	CAACACTTCATACTGGCCACTTTGCCCAT	79	78		Etv6	NM_007961.3:3859
62	Ezh2	NM_007971.2	426-525	AGTCATCCCGTTAAAGACCCTGAATGCAG	83	79		Ezh2	NM_007971.2:425
63	Fas	NM_007987.2	96-195	GGCTCACAGTTAAGAGTTCATACTCAAGG	81	82		Fas	NM_007987.2:95
64	Fcgr2b	NM_001077189.1	1226-1325	TTGGTTCCCAATGGTTGACTGTACTAATGA	79	80		Fcgr2b	NM_001077189.1:1225
65	Fcgr3	NM_010188.5	1176-1275	TCTGACCTCCACCATCCACCATGGCAGGT	82	81		Fcgr3	NM_010188.5:1175
66	Fli1	NM_008026.4	1866-1965	AGCAGTTTCTTATCAGCACACGGGTCTCA	78	80		Fli1	NM_008026.4:1865
67	Flt3	NM_010229.2	3303-3402	CGCCCTACAGGCCGTTGCTTCGCTGGACT	82	80		Flt3	NM_010229.2:3302
68	Fosl1	NM_010235.2	354-453	AGCAGAAGTTCCACCTTGTGCCAAGCATC	86	85		Fosl1	NM_010235.2:353
69	Foxo1	NM_019739.2	2531-2630	TTTCCTCAGACTTGGCAACAGCGGCAGCA	81	82		Foxo1	NM_019739.2:2530
70	Foxo3	NM_019740.2	2222-2321	GATGCTGACGGGTTGGATTTTAACTTTGA	80	82		Foxo3	NM_019740.2:2221
71	G6pdx	NM_008062.2	2031-2130	ACATTCTAGTTCCTGGGCTTGGACCGCCA	82	83	HK	G6pdx	NM_008062.2:2030
72	Gapdh	NM_008084.2	522-621	CTGGCCAAGGTCATCCATGACAACTTTGG	85	86	HK;X	Gapdh	NM_008084.2:521
73	Gata1	NM_008089.1	1641-1740	TTACAGGCATGTGCTACCGTGCCCGTTTC	81	80		Gata1	NM_008089.1:1640
74	Gata2	NM_008090.4	2961-3060	AGGGAGACGATTGTGCTGAGTCAACCAAG	82	82		Gata2	NM_008090.4:2960
75	Gata3	NM_008091.3	1944-2043	CATGCGTGAGGAGTCTCCAAGTGTGCGAA	83	80		Gata3	NM_008091.3:1943
76	Gfi1	NM_010278.2	1876-1975	CAAAGCTCATCATGGTTAGTCCCCTTCAC	81	82		Gfi1	NM_010278.2:1875
77	Gfi1b	NM_008114.3	1095-1194	CAGCGCAAGGTGGACCTGCGACGTCACC	82	82		Gfi1b	NM_008114.3:1094
78	Gusb	NM_010368.1	1736-1835	AATACGTGGTCGGAGAGCTCATCTGGAAT	79	81	HK	Gusb	NM_010368.1:1735
79	Hes5	NM_010419.4	1146-1245	ATCCTTAAAGGATTCCTTTGTATGGGTGG	78	80		Hes5	NM_010419.4:1145
80	Hlf	NM_172563.3	2141-2240	TTCCACCAACCCCAGATAAGTAAGTACCA	81	79		Hlf	NM_172563.3:2140
81	HPRT	NM_013556.2	31-130	TGCTGAGGCGGCGAGGGAGAGCGTTGGG	82	82	HK	Hprt	NM_013556.2:30
82	Id2	NM_010496.3	626-725	AAGAAAGCGGAAGGAAAACTAAGGATGAT	83	80		Id2	NM_010496.3:625
83	Ifi203	NM_001302649.1	2183-2282	CGAAAAGGATGTTTCATGCTACAGTGGCT	79	80	X	Ifi203	NM_001302649.1:2182
84	Ifi205	NM_172648.3	42-141	TGAATTATCTGCCTACCTACTCAAACCAAG	81	82		Ifi205	NM_172648.3:41
85	Ifitm1	NM_001112715.1	413-512	CCTCCACCGCCAAGTGCCTGAACATCAGC	83	79		Ifitm1	NM_001112715.1:412
86	Ikzf1	NM_009578.2	1717-1816	ATGTTAGTGACAGGATTGCATTGCATCAG	79	79		Ikzf1	NM_009578.2:1716
87	Ikzf2	NM_011770.4	7231-7330	AATTATGGAACTGTGATCTAAAAACGGTTT	83	83		Ikzf2	NM_011770.4:7230
88	Il7R	NM_008372.3	1021-1120	GTTTCCTGGACTGCCAGATTCATGAGGTG	82	80		Il7r	NM_008372.3:1020
89	Irf4	NM_013674.1	1879-1978	AAGGGACTTGGTTCTTAGGTCCAGTCTAG	82	81		Irf4	NM_013674.1:1878
90	Irf6	NM_016851.2	2411-2510	TTGATGAGCAACTATGATTCCATGTGCCTC	82	79		Irf6	NM_016851.2:2410
91	Irf8	NM_008320.3	2275-2374	GCAGGGAGAGTCATCATCTGCTTTTGTCT	82	79		Irf8	NM_008320.3:2274
92	CD41	NM_010575.2	462-561	CCCGTTGCTCTTCGACCTCAGGGATGAGA	85	85		Itga2b	NM_010575.2:461
93	LSD1	NM_133872.1	1264-1363	AACAAGAATTTAACCGGTTGCTAGAAGCC	79	79		Kdm1a	NM_133872.1:1263
94	Kdr	NM_010612.2	996-1095	TTAAATTGTACAGCGAGAACAGAGCTCAA	81	81		Kdr	NM_010612.2:995
95	Kit	NM_001122733.1	4276-4375	CAGTTTCCCTGCATGTCGCTCACTGTCTA	80	83		Kit	NM_001122733.1:4275
96	Klf1	NM_010635.2	1107-1206	TGCTGTGGCCTCTGCCCACGTGCTTTTTC	81	83		Klf1	NM_010635.2:1106
97	Klf12	NM_010636.3	4776-4875	TGAATAGATCCGGTGAGGGGAGGCCTTCA	82	78		Klf12	NM_010636.3:4775
98	Mll5	NM_026984.1	881-980	TGCAGTGTTTGGCAACATATTGACTGCAT	82	81		Kmt2e	NM_026984.1:880
99	Ldb1	NM_010697.1	1051-1150	AACCCATGTTTACCCAGGTGTGTGTGGAA	82	82		Ldb1	NM_010697.1:1050
100	Lin28a	NM_145833.1	2741-2840	AAGGACCCATGTGAAAATTCTGAAGAAGC	80	81		Lin28a	NM_145833.1:2740
101	Lmo2	NM_001142335.1	825-924	GTGGGTGAGGCATTTCTTAGGAGATTATG	83	83		Lmo2	NM_001142335.1:824

	Gene	Accession	Position	Target Sequence	Tm CP	Tm RP	Flags	HUGO Gene	NSID (internal identifier used by NanoString)
102	Ly6a	NM_010738.2	207-306	GAGGCAGCAGTTATTGTGGATTCTCAAAC	80	81		Ly6a	NM_010738.2:206
103	Lyl1	NM_008535.2	1475-1574	TAGCTCTGGAAGGGTGACCGGCGACGCC	86	85		Lyl1	NM_008535.2:1474
104	Max	NM_008558.2	1081-1180	CCTCCCAGTCCTCCCTCACCTCTGTTTGG	82	82		Max	NM_008558.2:1080
105	Meis1	NM_010789.3	1264-1363	GTTTGAAAGGGAAAATGCCTATCGATTTG	79	82		Meis1	NM_010789.3:1263
106	Mllt3	NM_027326.3	4507-4606	AGCACATGATCTGAAAATACAGAAGTCAT	81	81		Mllt3	NM_027326.3:4506
107	Mpl	NM_001122949.1	296-395	ATGTGTGCCAGTTTCCAGCCCAGGATGAA	83	83		Mpl	NM_001122949.1:295
108	Myb	NM_010848.3	1745-1844	CCTACAAGATGTGATTAAGCAGGAATCGG	82	82		Myb	NM_010848.3:1744
109	Myc	NM_010849.4	631-730	CCCTCAACGTGAACTTCACCAACAGGAAC	83	78		Myc	NM_010849.4:630
110	Mycn	NM_008709.3	2081-2180	AAATACCATTGATACACCCGCCTTTTGTAT	80	83		Mycn	NM_008709.3:2080
111	Nfat5	NM_133957.3	1095-1194	AGAGGCCGGGGGTCAAACGACGAGATTG	84	82		Nfat5	NM_133957.3:1094
112	Nfia	NM_010905.3	8626-8725	TGTGAAGATTCCTGTCTCTCCCGTGGTTTT	79	80		Nfia	NM_010905.3:8625
113	Nfkb1	NM_008689.2	2126-2225	GTCTTACACTTAGCCATCATCCACCTCCA	83	81		Nfkb1	NM_008689.2:2125
114	Notch1	NM_008714.2	1426-1525	TGAGATTGATGTTAATGAGTGCATCTCCA	81	82		Notch1	NM_008714.2:1425
115	Oaz1	NM_008753.4	810-909	CCCTGTGCCCTCTCCTGGGTTAGTCCACA	83	82	HK;X	Oaz1	NM_008753.4:809
116	Pax5	NM_008782.2	91-190	TGAATCAGCTTGGGGGGGTTTTTGTGAAT	82	83		Pax5	NM_008782.2:90
117	Pbx1	NM_008783.2	952-1051	TGCTTTAAACTGCCACAGAATGAAGCCTG	77	82		Pbx1	NM_008783.2:951
118	Pecam1	NM_008816.2	1101-1200	ACACCTGCAAAGTGGAATCAAACCGTATC	82	80		Pecam1	NM_008816.2:1100
119	PIk3ca	NM_008839.1	1256-1355	ACTGTCCGTTGGCCTGGGGAAACATAAAC	81	82		Pik3ca	NM_008839.1:1255
120	PIk3R2	NM_008841.2	231-330	CCTATCCCAGCTTGACCATCTGATGGGTG	83	82		Pik3r2	NM_008841.2:230
121	Polr1b	NM_009086.2	2796-2895	TGCCTTTCACTGAGAGTGGCATGATGCCG	81	83	HK	Polr1b	NM_009086.2:2795
122	Pten	NM_008960.2	5161-5260	CAGCCTTACCCCGATTCAGCCTCTTCAGA	81	79		Pten	NM_008960.2:5160
123	Rag1	NM_009019.2	1946-2045	GAAGCACGGGAGTGGGCCCGCAGTTCCA	80	78		Rag1	NM_009019.2:1945
124	Rag2	NM_009020.3	1319-1418	GAAGATCCTGGGGACTCCACTCCCTTTGA	81	80		Rag2	NM_009020.3:1318
125	Rb1	NM_009029.2	1591-1690	CTGGCCTGTGCTCTTGAAGTTGTAATGGC	80	78		Rb1	NM_009029.2:1590
126	Rora	NM_013646.1	1046-1145	GTGTCAAAATGATCAAATTGTGCTTCTAAA	81	79		Rora	NM_013646.1:1045
127	Runx1	NM_001111021.1	3056-3155	ACAGTGGAGGACTTGGTCAAAATCCAGTT	82	79		Runx1	NM_001111021.1:3055
128	Runx2	NM_009820.5	1117-1216	TCCTATGACCAGTCTTACCCCTCCTATCTG	85	85		Runx2	NM_009820.5:1116
129	Satb1	NM_009122.1	2462-2561	GCACAGACGTTAATGCCGACTTGAAAGAC	78	82		Satb1	NM_009122.1:2461
130	Sdpr	NM_138741.1	159-258	TAGCCCTAGAGAGCAGTGCTCACTTCAGA	82	82		Sdpr	NM_138741.1:158
131	Sell	NM_001164059.1	665-764	GTCCAAGTGTGCTTTCAACTGTTCTGAGG	81	82		Sell	NM_001164059.1:664
132	Slamf1	NM_013730.4	1771-1870	AAGTGTGGTTCTCTGATTAAGTCAAAGCA	80	78		Slamf1	NM_013730.4:1770
133	Smarca4	NM_011417.2	3541-3640	CAACGAACCATAAAGTGCTCCTCTTTTGC	83	82		Smarca4	NM_011417.2:3540
134	Sos1	NM_009231.2	6003-6102	GACAGCCATCAGTATGTGCATGTGTCCTC	82	78		Sos1	NM_009231.2:6002
135	Sfpi1	NM_011355.1	201-300	TCGGATGACTTGGTTACTTACGATTCAGA	79	83		Spi1	NM_011355.1:200
136	Stat1	NM_009283.3	1591-1690	ACGCTGGGAACAGAACTAATGAGGGGCC	82	80		Stat1	NM_009283.3:1590
137	Stat3	NM_213659.2	2131-2230	GGGGTCACTTTCACTTGGGTGGAAAAGGA	81	81		Stat3	NM_213659.2:2130
138	Stat4	NM_011487.4	1817-1916	TAGAGACCAGCTCATTACCTGTCGTGATG	79	79		Stat4	NM_011487.4:1816
139	Stat6	NM_009284.2	3466-3565	CATTGTATAAGACAGCAACCCAGTATCATT	81	81		Stat6	NM_009284.2:3465
140	Suz12	NM_199196.1	821-920	TGTCCAATAAGACAAGTCCCTACTGGTAA	82	79		Suz12	NM_199196.1:820
141	Tal1	NM_011527.2	2491-2590	CCGATCTATCCTAAAGCTAGGCCTGCCTA	82	79		Tal1	NM_011527.2:2490
142	tbp	NM_013684.3	71-170	GTGGCGGGTATCTGCTGGCGGTTTGGCT	83	79	HK	Tbp	NM_013684.3:70
143	e2a	NM_001164147.1	704-803	GAGCGGAATGCCTATGCCACCTTTGGGAG	90	91		Tcf3	NM_001164147.1:703
144	TCF7	NM_009331.3	1811-1910	CTTTCCCAAGAAGCTCACCAGCATTAACA	79	81		Tcf7	NM_009331.3:1810
145	Tgfb1	NM_011577.1	1471-1570	GGAGTTGTACGGCAGTGGCTGAACCAAG	82	78		Tgfb1	NM_011577.1:1470
146	Tnfrsf1a	NM_011609.2	616-715	CTCCTTGCCAAGCTGACAAGGACACGGTG	82	82		Tnfrsf1a	NM_011609.2:615
147	Tnfrsf1b	NM_011610.3	3271-3370	GTGTGTGTCCATGTTTGCATGTATGTGTG	82	83		Tnfrsf1b	NM_011610.3:3270
148	Trp53	NM_011640.1	1836-1935	CCCTCTCTGAGTAGTGGTTCCTGGCCCAA	82	83		Trp53	NM_011640.1:1835
149	vWF	NM_011708.3	6355-6454	GTCAGCATCTACGGCGCTATCATGTATGA	79	82		Vwf	NM_011708.3:6354
150	Zbtb20	NM_019778.2	1890-1989	CCGATAAGGGCGTCCTACAGCAGCCTTCA	83	82		Zbtb20	NM_019778.2:1889

Supplementary Table 2. List of Taqman primer probes

Gene Name	Gene Assay ID (in Applied Biosystems)
Stat3	Mm01219775_m1
Ikzf1	Mm01187877_m1
Runx1	Mm01213404_m1
Cdk1	Mm00772472_m1
Csf1r	Mm00432689_m1
Spi1	Mm00488140_m1
Id2	Mm00711781_m1
Lgr4	Mm00554385_m1
Runx2	Mm00501580_m1
Notch1	Mm00627185_m1
Flt3	Mm00439016_m1
Cdkn2b	Mm00483241_m1
Tcf3	Mm01175588_m1
Ebf1	Mm00432954_m1
Stat4	Mm00448890_m1
Actb	Mm00607939_s1
